# Prevalence and Associated Factors of Suicide Ideation and Attempt among Adolescent High School Students in Dangila Town, Northwest Ethiopia

**DOI:** 10.1155/2018/7631453

**Published:** 2018-06-11

**Authors:** Tadele Amare, Solomon Meseret Woldeyhannes, Kelemua Haile, Tebikew Yeneabat

**Affiliations:** ^1^Department of Psychiatry, College of Medicine and Health Science, University of Gondar, Gondar, Ethiopia; ^2^Department of Public Health, College of Medicine and Health Science, University of Gondar, Gondar, Ethiopia; ^3^Department of Psychiatry, Amanuel Mental Specialized Hospital, Addis Ababa, Ethiopia; ^4^Department of Midwifery, College of Health Sciences, Debre Markos University, Debre Markos, Ethiopia

## Abstract

**Background:**

Suicide is a major public health problem and is common among adolescents worldwide. The true extent of the problem in Ethiopia is difficult to ascertain as suicides and suicidal behavior are significantly underreported and understudied.

**Objective:**

We assessed the prevalence and factors associated with suicide ideation and suicide attempt among adolescent high school students in Dangila Town, Ethiopia.

**Methods:**

This school-based cross-sectional study was conducted from April to May 2015 in Dangila Town. Data were collected from adolescent high school students using pretested, self-administered Amharic-language questionnaire. We conducted bivariable and multivariable logistic regression to identify the independent factors associated with suicide ideation and attempt.

**Results:**

A total of 573 of 603 sampled students participated in the study (95% response rate). The mean (±SD) age of the respondents was 17.52 (±0.97) years. The minimum and maximum ages were 15 and 19 years, respectively. The prevalence of suicide ideation and attempt was 22.5% and 16.2%, respectively. School absenteeism [AOR 4.30, 95% CI (2.03, 9.10)] and poor social support [AOR 5.58, 95% CI (2.25, 13.84)] were positively associated with suicide ideation. Poor social support [AOR 4.55, 95% CI (1.40, 14.77)] and being physically hurt [AOR 4.25, 95% CI (1.77, 10.20)] were positively associated with suicide attempt. Unlike previous studies of adolescents in low-income countries, we find no association between gender or alcohol use and suicidal thoughts or attempts.

**Conclusion:**

This study revealed that at least one in five of the adolescents in our sample had experienced suicide ideation and one in six had attempted suicide. School absenteeism, poor social support, and experience of violence were identified as independent contributors to suicide ideation and attempt. These findings suggest a need for education policymakers to implement school-based behavioral therapy programs in collaboration with health institutions and programs to provide social support for vulnerable students.

## 1. Background

Suicide (from Latin Sui caedere to kill oneself or self-murder) means the act of a person intentionally causing his or her own death. It is death from injury, poisoning, or suffocation where there is evidence (either explicit or implicit) that the injury was self-inflicted and that the decedent intended to kill himself/herself. The suicide attempt is a nonhabitual act with the nonfatal outcome that is deliberately initiated and performed by the individual involved that causes self-harm or without intervention by others will do so or consists of ingesting a substance in excess of its generally recognized therapeutic dosage [1–5]. Suicide ideation is any self-reported passive thought about wanting to be dead or active thoughts about killing oneself not accompanied by preparatory behavior [1–4].

Suicide accounts for approximately 1.4% of the global burden of disease and is the second leading cause of death in adolescents in globally. Each year more people died from suicide than from all homicide and wars combined. Although suicide rate increases with age, suicidal behavior is high and is increasing among youth people between the ages of 15 and 25 years. Over 800,000 young people in this age group die each year from suicide [6–8].

Although most of the research on suicide attempt and ideation among adolescents has been conducted in high-income countries, there is a growing literature that explores this health threat in low- and middle-income countries. Studies in Asia and the Middle East reveal a high prevalence of suicide ideation and attempt [9, 10]. In these studies rates of ideation ranged from 6% in India [11] to 25.28% in Palestine in lifetime [12]. The prevalence of suicide attempt ranged from 0.39% in India [11] to 2.7% in China [13] to 3.8% in Vietnam in lifetime [14].

In sub-Saharan Africa, death from suicide is estimated to be 34,000 per year [15]. Studies have found high rates of suicide ideation in the last 12 months among high school students. The prevalence of suicide ideation was estimated to be 7% in Tanzania [16], 6.2% in Seychelles [17], 18.4% in Guyana [18], 31.3% in Zambia [19], 23.3% in Benin [20], and 21.6% in Uganda [21]. In Ethiopia, suicide ideation is not uncommon and that it seems to be more common among men than women [22].

The factors found to be associated with suicide ideation among young people in Africa were female gender [20, 23], loneliness [10, 16, 17, 20, 21], sadness [10, 16, 19, 23], and anxiety [17, 20, 21]. For example, anxiety was present in 51.4% of individuals who reported suicide ideation in Benin [20]. Alcohol use has been found to be associated with suicidal ideation in Zambia [19] and substance use with ideation in Tanzania [16], Seychelles [16], and Benin [20].

Suicide attempt among youth between the ages 15 and 24 has also been found to be relatively common on the continent, ranging from 12% of the study respondents in Southwest Nigeria to 28.3% in Benin in the last one year [20, 23–25]. There is relatively sparse information on the drivers of suicide attempt among adolescents in low-income countries. A study done in China [13] revealed scolding by parents, the experience of punishment, and family gambling were associated with suicide attempt. Loneliness, feeling depressed, tobacco use, and having no friends were associated with suicide attempt in Tanzania [16].

Despite these scattered studies, the true extent of the problem in Ethiopia is poorly understood, as there have been very few studies in the country and statistics on suicide are not significantly understood [26]. This study begins to address this gap by determining the magnitude and associated factors of suicide ideation and attempt among adolescent high school students.

Suicide is a serious, preventable public health problem that results in social, emotional, and economic consequences in families, friends, and colleagues [1, 6]. Suicide demands our attention and action even though its prevention and control are difficult [27, 28]. We hope that the evidence generated in this study will facilitate these efforts.

## 2. Methods

### 2.1. Study Design and Setting

This school-based, cross-sectional study was conducted from April 2015 to May 2015 in Dangila Town. Dangila is the town of Dangila Woreda found in the Awi Zone of the Amhara Regional State in Ethiopia. It is located at 485 km away from the capital city, Addis Ababa. There are two high schools and one preparatory school in the town of 2100, 2397, 1044, and 855 students in grades 9, 10, 11, and 12, respectively. The total number of students enrolled in high school during data collection was 6396 (3140 males and 3256 females).

The source population for the study was all adolescent students in Dangila Town high schools enrolled in 9th to 12th grade in 2014/2015. The sample size required for this study was 603, determined by using single population proportion formula [29] that assumes a 14.3% prevalence [24] and sets a 3% margin of error and 15% nonresponse rate. We used a simple random sampling technique to select the participants from each of the town's three high schools based on their student identification number. We combined student lists from each school and selected participants using computer-generated random numbers.

We collected data using a self-administered, Amharic-language questionnaire containing both closed-ended and open-ended questions and validated standardized scales. The questionnaire contained questions on lifetime suicide ideation (whether an individual had ever thought of killing himself or herself in his or her lifetime) and lifetime suicide attempt (whether an individual had ever tried to kill himself or herself in his or her lifetime). In addition, we included questions on sociodemographic characteristics, clinical factors, psychosocial characteristics, and substance abuse, all of which were considered to be potential drivers of suicide ideation and attempt. The study questionnaires were adapted from the World Mental Health survey initiative version of the World Health Organization Composite International Diagnostic Interview (CIDI) [30], which is a standard tool used to assess depression and the prevalence and associated factors of suicide ideation and attempt. It also contained a three-item Oslo Social Support Scale [31], which is used to assess social support. CIDI sections of our questionnaire included questions on mood disorders and substance use disorders among others.

The tool was pretested two weeks prior to data collection on 5% of the sample (31 students) at Adis Kidam High school. The internal consistency of the questionnaire was determined using Cronbach's alpha (alpha = 0.71) reliability statistical test. Trained Bachelor of Science Nurses collected the data. They provided brief instructions to participants on how to respond to the questionnaire and collected completed questionnaires the same day.

### 2.2. Data Processing and Analysis

After collection, we checked the surveys for completeness and consistency and then coded and entered responses using EPI info version 3.5.3 software. We conducted our analysis using SPSS version 20. We generated descriptive statistics to describe our variables of interest and to estimate the prevalence of suicide ideation and attempt. We conducted bivariable and multivariable logistic regression to identify factors associated with suicide ideation and attempt. The strength of association was estimated using the crude and adjusted odds ratio (COR and AOR) with 95% confidence interval (CI). Independent variables that were associated with outcomes at the *p* < 0.2 level in the bivariable logistic regression were included in multivariable logistic regression models using the enter method. Variables that were significant at the *p* < 0.05 level in the multivariable logistic regression are reported as independent factors associated with dependent variables.

### 2.3. Operational Definition

- Adolescent defined as individuals between 10 and 19 years of age.
- Social support was categorized based on the previous study [32] as follows:
  - Poor social support defined as when individual scores “3–8” based on Oslo Social Support Scale
  - Moderate social support defined as when individual scores “9–11” based on Oslo Social Support Scale
  - Strong social support defined as when individual scores “12–14” based on Oslo Social Support Scale
- Substance (alcohol, khat, and cigarette) use was categorized based on the previous study [33] as follows:
  - Ever substance use defined as consuming any substance at least once in his or her lifetime
  - Current substance use defined as consuming any substance at least once in the last month
- WHO defines adolescence as individuals between 10 and 19 years of age [34].
- Having seen adults physically hurting another adult in the last 12 months is defined as the case when the individual saw an adult in the family members physically hurting another adult (father, mother, sister, brother, and any family members)
- Having seen adults physically hurting a child in the last 12 months is defined as the case when individual saw adults in the family members physically hurting a child.

### 2.4. Ethical Consideration

Ethical clearance was obtained from the Institution Review Board (IRB) of the College of Medicine and Health Science, University of Gondar, and from the Amanuel Mental Specialized Hospital. Permission was obtained from the Dangila Town Education Office. The aims of the study were explained for the study participants and data were collected after assent and written consent. Students aged 18 years and above gave informed written consent to participate. Students under the age of 18 gave verbal assent to the study and then written consent was obtained from their parents/caretakers/guardians/kin on their behalf. Both students and parents were informed that they had the right to refuse to answer any question at any time, that they had the opportunity to ask questions about the study both during and after the study, and that their confidentiality was assured. The survey was anonymous and no identifying information was collected. Our data management procedures assured the confidentiality of the collected data.

## 3. Results

### 3.1. Sociodemographic Characteristics of the Respondents

Of the 603 sampled students, 573 (95%) students completed our questionnaire. The remaining 30 students did not respond to all of the questions and were, therefore, excluded from the analysis. The mean (±SD) age of the respondents was 17.52 (±0.97) years. The minimum and maximum ages were 15 and 19 years, respectively. The majority (96%) of respondents were unmarried. Of the married students, ten were living with their spouse, 11 with their family, and two were alone. More details on the sociodemographic characteristics of the sample are listed in Table 1.

### 3.2. Psychosocial and Substance Related Factors

We found that 68.8% of the surveyed students (394) were disappointed by failing school results, and 11.7% (67) had been absent from school without permission for more than three days in the last month. We found a surprisingly large proportion of students reporting poor social support but low levels of abuse and violence (Table 2).

Approximately one-fourth (25.1%) of study participants reported feeling loneliness, 14.8% (85) feeling hopelessness/sadness, and 32.1% (184) being worried about something so much that they could not sleep at night. Almost 4% (21) of respondents had a family history of suicide attempt.

Almost a third of participants reported having drunk alcohol in their lifetime and a little more than a one-fifth reported current usage. Khat and tobacco use was more modest; 5.2% had chewed khat and 4.0% had smoked cigarette (Table 3).

### 3.3. Prevalence of Suicide Ideation and Attempt

The prevalence of lifetime suicide ideation was 22.5% (95% CI: 19.4%, 26.3%). Among those who had the history of suicide ideation, 34 (26.36%) had thought about committing suicide in the last one month and 31 (24.03%) had planned to commit suicide in their lifetime.

The prevalence of lifetime suicide attempt was 16.2% (95% CI: 13.4%, 19.4%). Among these respondents, the proportion that had attempted suicide in the last month was 19.35%. Of the respondents who had ever attempted suicide, 20.43% had two attempts and 15.05% had tried more than twice in their lifetime. Of those who had attempted suicide in their lifetime, 64.52% had made one attempt, 20.43% had made two attempts, and 15.05% had made more than two attempts (Table 4).

**Figure 1:**
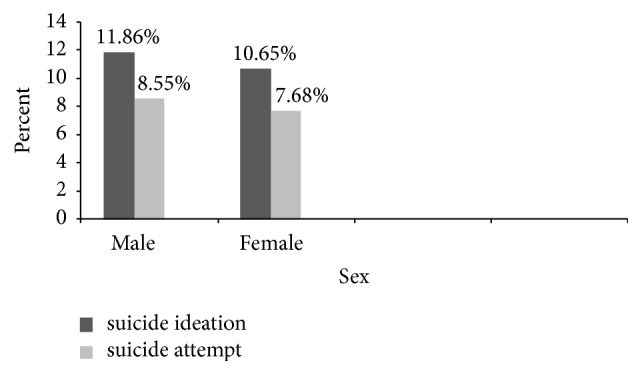
Lifetime suicide ideation and suicide attempt by gender among adolescent high school students in Dangila Town, Northwest Ethiopia, 2015.

In this study both suicide ideation and attempt were relatively higher among boys compared to girls (Figure 1).

**Figure 2:**
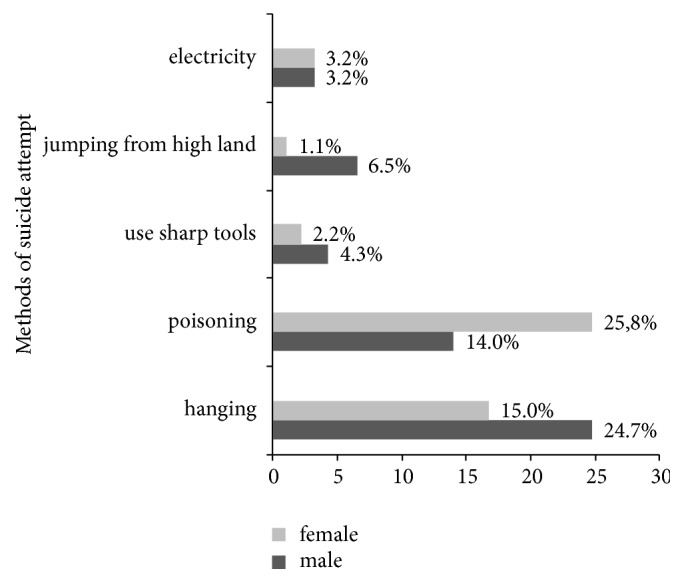
Distribution of suicide attempt methods by sex among adolescent high school students in Dangila Town, Northwest Ethiopia, 2015.

Of those who attempted suicide, five methods of suicide attempt were used. Hanging was more used by the boys (23, 24.7%) and poisoning was more used by the girls (24, 25.8%). Electricity and use of sharp tools in the suicide attempt were less frequent (Figure 2).

**Figure 3:**
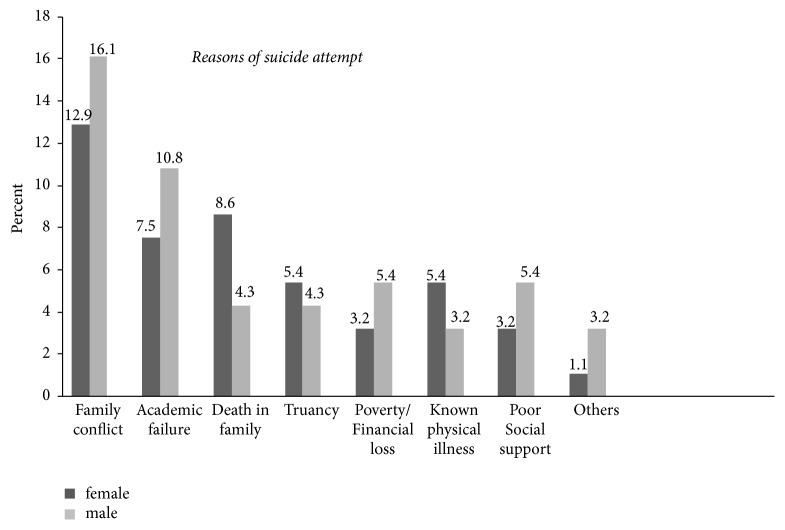
Reasons of lifetime suicide attempt among adolescent high school students in Dangila Town, Northwest Ethiopia, 2015.

Concerning the seriousness of suicide attempt, 37.6% of study participants report having attempted suicide to “cry for help” and 30.1% report a serious attempt to kill themselves and it was only luck that they did not succeed. Respondents gave a variety of reasons for their suicide attempt, the most frequent being family conflict, academic failure, and death in the family. A death in the family, truancy, and known physical illness were more frequently cited as reasons for suicide attempts for the young women in the study than the young men (Figure 3).

### 3.4. Factors Associated with Lifetime Suicide Ideation among Adolescent Students

Students who were disappointed with their school results had twice the odds of reporting suicide ideation [AOR = 2.23] than their peers. Those who had been absent from school for more than three days in the last month had approximately four times the odds [AOR = 4.30] of suicide ideation. Those who felt lonely [AOR = 2.42] and hopeless/felt sad [AOR = 4.66] were more likely to report suicide ideation. Adolescents who had poor social support were about 5.6 times [AOR = 5.58] more likely to report suicide ideation than participants who had strong social support. The odds of having suicide ideation was about 4.9 times [AOR = 4.85] higher among those who have been physically hurt as compared to their counterparts (Table 5).

### 3.5. Factors Associated with Suicide Attempt among Adolescent Students

Adolescent students who had been living alone had about twice the odds [AOR = 2.08] of attempting suicide compared to those who had been living with their family. As with ideation, school absenteeism, loneliness, and feelings of hopeless and sadness were significantly positively associated with suicide attempt in bivariable and multivariable regression [AOR = 2.41, AOR = 2.46, and AOR = 4.51, respectively]. The odds of suicide attempt were more than twice [AOR = 2.32] as high among students who had sleep disturbance in the last 12 months and four times greater [AOR = 4.25] among those who reported being physically hurt in the last 12 months. Those who had poor social support had 4.6 times the odds of attempting suicide compared to those who had strong social support [AOR = 4.55] (Table 6).

## 4. Discussion

This study finds a high prevalence of lifetime suicide ideation and attempt. Our lifetime prevalence of suicide ideation (22.5%) is consistent with findings from Vietnam (26.3%) [14] but is much higher than the rates found in India (6%) [11]. The inclusion of individuals aged less than 15 years in the India study might have a contribution to the lower prevalence found there as evidence points to the peak of suicide ideation occurring after 15 years of age [8, 24]. The difference in ideation rates might also be due to the difference in measurement tools used: CIDI [30] was used in this study, whereas a general health questionnaire was used in India [11].

Several factors were associated with lifetime suicide ideation in this study, most of them in keeping with previous studies. For example, the odds of suicide ideation was 2.2 times higher among students disappointed with failing school results compared to those who were not disappointed [AOR = 2.23, 95% CI (1.15, 4.35)]. Previous studies [34, 35] also have found poor school performance is positively associated with suicide. The positive association between truancy and suicide ideation is in keeping with findings from Thailand [10] and Benin [20]. As with studies in Seychelles [17], Tanzania [16], Benin [20], and Rural Uganda [21] we also find strong positive associations between loneliness and suicide ideation and our findings of positive associations between feelings of hopelessness and sadness and contemplating suicide were in keeping with those reported in Thailand, Seychelles, Tanzania, Zambia, and South Africa [10, 16, 17, 19, 23]. As other studies have found, our respondents were more likely to report contemplating suicide when they lacked peer support [10] and had no close friends [17] and if they had been physically hurt in the last 12 months [10, 16, 20]. However, unlike other sub-Saharan African studies, a family history of suicide, alcohol intake, khat chewing, and cigarette smoking were not associated with suicide ideation [16, 20].

The prevalence of suicide attempt in this study (16.2%) is almost the same as the previous study done in Ethiopia (14.3%) [24] but is markedly higher than those found in Korea (3.3%) [36], Vietnam (3.8%) [14], India (0.33%) [11], and Nigeria (12%) [25]. This discrepancy might be attributable to small sample sizes in other studies (e.g., *n* = 368 in Korea); differences in study settings and sociocultural characteristics; and differences in the suicide attempt variable; for example, the study in Nigeria examined suicide attempt in the last year only, not lifetime rates as we did. Additionally, the Indian study included participants aged less than 15 years; an age group in which suicide attempts exhibit relatively low prevalence. Once again, the difference in reported suicide attempt rates might also be attributed to the difference in measurement tools used.

Adolescents who live alone have about two times the odds of reporting compared to those who live with their families. Living together with families and/or peers encourages adolescents to share their feelings and thoughts especially during stressful events, which could reduce emotional or mental disturbance. Those living alone do not have the opportunity to share their feelings and social support is poor, which may be stressful leading to suicide attempts [37]. In line with this reasoning, those who felt lonely in the last 12 months were about 2.5 [AOR 2.46, 95% CI (1.29, 4.70)] times more likely to experience suicide attempt. Moreover, adolescents who had poor social support were 4.6 [AOR = 4.55, 95% CI (1.40, 14.77)] times more likely to have tried suicide than individuals who had strong social support. This was similar to results from a recent study in China [13]. This might be because individuals with poor social support feel helpless, particularly if they live alone. Social support was found to be a significant factor in suicide attempt in the previous study of adolescents in low-income countries [12].

Feeling hopeless or sad in the last 12 months increased the odds of attempting suicide by 4.5 [AOR = 4.51, 95% CI (2.24, 9.08)] times. This is similar to findings from previous research done in Ethiopia [24]. The odds of having suicide attempt was 2.3 [AOR = 2.32, 95% CI (1.25, 4.28)] times higher among those worried about their sleep disturbance in the last 12 months as compared to not being worried. This finding is supported by the study done in New Zealand [38].

Participants who reported being physically hurt in the last 12 months were 4.3 [AOR = 4.25, 95% CI (1.77, 10.20)] times more likely to attempt suicide compared to their counterparts. This finding is supported by similar results from a study done in New Zealand [38]. The possible reason might be for escape from suffering in physical injury. The possible explanation might be for escape from suffering in physical injury. It has been shown that family conflict is associated with suicidal behavior [39].

## 5. Limitations

This study is not without its limitations. Because it is cross-sectional, the lifetime frequency of suicide ideation and attempt could not be collected due to recall bias and causal relationships cannot be established between independent factors and suicide ideation and attempt. Another limitation is the fact that we were not able to study adolescents out of school. This study was conducted in one town in Ethiopia, which limits its generalizability to other settings. However, it is one of the few studies that has been conducted on adolescent suicide ideation and attempt in Ethiopia and so, we believe, it makes an important contribution to the literature.

## 6. Conclusion

This study revealed that suicide ideation and attempt were common among adolescents in high school. We find that Ethiopian adolescents behave relatively similar to their counterparts in other low-income countries and that the psychosocial factors that have been found to be associated with suicide ideation and attempts in other settings are also significant factors in Ethiopia. School absenteeism, abuse, and psychosocial distress were identified as independent factors predicting suicide ideation and attempt. This suggests that schools programs to provide psychosocial support and to encourage students to work in groups (peer academic support) to improve academic achievement might be important strategies to reduce suicide ideation and attempt. Mental health modules should also be included in the high school curriculum. Finally, further research is needed to examine suicide ideation and attempt among adolescents out of school.

## Figures and Tables

**Table 1:** Distribution of sociodemographic characteristics of adolescent high school students in Dangila Town, Northwest Ethiopia, 2015.

Variables	Frequency	Percent
Sex		
Female	296	51.7
Male	277	48.3
Age		
<18 years	251	43.8
18 years	244	42.6
>18 years	78	13.6
Ethnicity		
Amhara	421	73.5
Awi	152	26.5
Marital status		
Single	550	96.0
Married	23	4.0
Religion		
Orthodox	556	97.0
Muslim	10	1.8
Protestant	7	1.2
Grade enrolled		
9th	184	32.1
10th	220	38.4
11th	90	15.7
12th	79	13.8
Living arrangement		
With family	431	75.3
With spouse	10	1.7
Alone	132	23.0

**Table 2:** Frequency distribution of psychosocial factors among adolescent high school students in Dangila Town, Northwest Ethiopia, 2015.

Variables	Number	Percent
Disappointed with school results in the last 12 months		
Yes	394	68.8
No	179	31.2
Absent from school greater than 3 days in the last month		
Yes	67	11.7
No	506	88.3
Social support		
Poor	201	35.1
Moderate	292	51.0
Strong	80	14.0
Saw adults physically hurting another adult in the last 12 months		
Yes	57	9.9
No	516	90.1
Being physically hurt in the last 12 months		
Yes	51	8.9
No	522	91.1
Saw adults physically hurting a child in the last 12 months		
Yes	36	6.3
No	537	93.7

**Table 3:** Frequency distribution of substance related factors among adolescent high school students in Dangila Town, Northwest Ethiopia, 2015.

Variables	Number	Percent
Ever use of alcohol drinking		
Yes	161	28.1
No	412	71.9
Ever use of khat chewing		
Yes	30	5.2
No	543	94.8
Ever use of cigarettes smoking		
Yes	23	4.0
No	550	96.0
Current use of alcohol drinking		
Yes	130	22.7
No	443	77.3
Current use of khat chewing		
Yes	22	3.8
No	551	96.2
Current use of cigarette smoking		
Yes	14	2.4
No	559	97.6

**Table 4:** Frequency distribution of suicide ideation and attempt among adolescent high school students in Dangila Town, Northwest Ethiopia 2015.

Variables	Frequency	Percent
Lifetime suicide ideation		
Yes	129	22.5
No	444	77.5
Suicide ideation in the last one month		
Yes	34	26.36
No	95	73.64
Lifetime suicide plan		
Yes	31	24.03
No	98	75.97
Lifetime suicide attempt		
Yes	93	16.2
No	480	83.8
Suicide attempt in the last one month		
Yes	18	19.35
No	75	80.65
The lifetime frequency of suicide attempt		
Once	60	64.52
Twice	19	20.43
More than twice	14	15.05

**Table 5:** The bivariable and multivariate logistic regression analysis results of suicide ideation and associated factors among adolescent high school students in Dangila Town, Northwest Ethiopia, 2015.

Variables	Lifetime Suicide ideation		COR (95% CI)	AOR (95% CI)
	Yes	No		
Disappointed with grade results in the last 12 months				
Yes	106	288	2.50 (1.53, 4.08)	**2.23 (1.15, 4.35)** ^*∗*^
No	23	156	1	1
School absence > 3 days in the last month				
Yes	39	28	6.44 (3.767, 11.01)	**4.30 (2.03, 9.10)** ^*∗∗∗*^
No	90	416	1	1
Lonely in the last 12 months				
Yes	74	70	7.19 (4.67, 11.08)	**2.42 (1.34, 4.38)** ^*∗∗*^
No	55	374	1	1
Hopeless/felt sad in the last 12 months				
Yes	58	27	12.62 (7.49, 21.25)	**4.66 (2.31, 9.43)** ^*∗∗∗*^
No	71	417	1	1
Social support				
Poor	90	111	6.40 (3.03, 13.50)	**5.58 (2.25, 13.84)** ^*∗∗∗*^
Moderate	30	262	0.90 (0.41, 1.99)	1.01 (0.39, 2.60)
Strong	9	71	1	1
Being physically hurt in the last 12 months				
Yes	32	19	7.38 (4.01, 13.57)	**4.85 (2.03, 11.59)** ^*∗∗∗*^
No	97	425	1	1
Drunk alcohol				
Yes	53	108	2.17 (1.44, 3.28)	1.24 (0.67, 2.29)
No	76	336	1	1

^*∗*^ *p* value is significant at *p* < 0.05; ^*∗∗∗*^*p* value is significant at *p* < 0.001; ^*∗∗*^*p* value is significant at *p* < 0.01.

**Table 6:** The bivariable and multivariable logistic regression analysis results of suicide attempt and associated factors among adolescent high school students in Dangila Town, Northwest Ethiopia, 2015.

Variables	Lifetime suicide attempt		COR (95% CI)	AOR (95% CI)
	Yes	No		
Living arrangement				
Family	53	378	1	1
Spouse	1	9	0.33 (0.21, 0.54)	0.53 (0.04, 7.44)
Alone	39	93	0.27 (0.03, 2.16)	**2.08 (1.09, 3.97)** ^*∗*^
Disappointed with grade results				
Yes	78	316	2.70 (1.51, 4.84)	2.08 (0.92, 4.48)
No	15	164	1	1
School absence > 3 days in the last month				
Yes	29	38	5.27 (3.04, 9.13)	**2.41 (1.12, 5.19)** ^*∗*^
No	64	442	1	1
Lonely in the last 12 months				
Yes	60	84	8.57 (5.27, 13.93)	**2.46 (1.29, 4.70)** ^*∗∗*^
No	33	396	1	1
Hopeless/felt sad in the last 12 months				
Yes	49	36	13.74 (8.08, 23.34)	**4.51 (2.24, 9.08)** ^*∗∗∗*^
No	44	444	1	1
Worried about sleep disturbance in the last 12 months				
Yes	61	123	5.53 (3.44, 8.89)	**2.32 (1.25, 4.28)** ^*∗∗*^
No	32	357	1	1
Social support				
Poor	62	139	6.69 (2.58, 17.36)	**4.55 (1.40, 14.77)** ^*∗*^
Moderate	26	266	1.47 (0.54, 3.95)	1.86 (0.55, 6.22)
Strong	5	75	1	1
Being physically hurt in the last 12 months				
Yes	26	25	7.06 (3.85, 12.95)	**4.25 (1.77, 10.20)** ^*∗∗*^
No	67	455	1	1
Drunk alcohol				
Yes	45	116	2.94 (1.86, 4.65)	1.75 (0.91, 3.35)
No	48	364	1	1

^*∗*^ *p* < 0.05; ^*∗∗*^*p* < 0.01; ^*∗∗∗*^*p* < 0.001.
